# Linear epitope prediction in HPV type 16 E7 antigen and their docked interaction with human TMEM 50A structural model

**DOI:** 10.6026/97320630013122

**Published:** 2017-05-31

**Authors:** Upasna Srivastava, Satendra Singh, Budhyash Gautam, Pramod Yadav, Madhu Yadav, George Thomas, Gurmit Singh

**Affiliations:** 1 Department of Computational Biology and Bioinformatics, Sam Higginbottom Institute of Agriculture, Technology and Sciences,Allahabad-211007, India; 2 Jacob School of Biotechnology and Bioengineering, Sam Higginbottom Institute of Agriculture, Technology and Sciences, Allahabad-211007, India; 3 Department of Computer Science and Information Technology, Sam Higginbottom Institute of Agriculture, Technology and Sciences, Allahabad-211007, India

**Keywords:** HPV, Cervical cancer, IEDB, Discovery Studio, Epitopes, TMEM 50

## Abstract

Human Papilloma Virus (HPV) HPV type 16 E7 antigen is a known target in cervical cancer. We report the predicted potential epitopes in the E7 antigen. We further describe the subsequent interaction of these linear epitope peptides with the human TMEM 50 A structural model using molecular docking. This data finds application in the development of components towards HPV associated disease prevention.

## Background

Human papillomavirus (HPV) are a group of viruses associated with various proliferative diseases [1]. HPV types 6, 11, 16, and 18 are the most common and are associated with lesions in the genital tract [2]. Several clinical, molecular, and epidemiological investigations have identified human papillomavirus (HPV) as the major cause of cervical cancer and cervical dysplasia [3]. HPV infection is limited to the epithelium so when the HPV particles have entered host cells, infection is dealt with cell-mediated immunity. Cytotoxic T-lymphocytes (CTLs) recognize foreign peptide antigens presented on the infected target cell surface by molecules of major histocompatibility complex (MHC). In the presence of co-stimulatory molecules binding to a CTL may induce an immune response. Infiltrating T-cells are seen in regressing warts caused by low-risk HPVs. Cervical cancer show tumor infiltrating lymphocytes that are predominantly CTLs [4]. Cervical cancer is also defined as the cancer originating at the opening of the womb, which progressively migrates to the whole of cervix [5]. It is inferred to be hereditary [6] but in 99.7% of cases, is caused by persistent infection with HPV. Some available forms of treatment such as surgery, radiation therapy and chemotherapy are all cyto-reductive treatment modalities. Healthy cells are also destroyed in addition to cancer cells in the process. Hence, there is a need to decrease the incidence of cervical cancer and develop better forms for its treatment [7]. Therefore, it is of interest to identify antigenic epitopes in HPV 16 E7 proteins and human TMEM 50A protein using advanced predictive models [8].

## Methodology

### Workflow

**Figure 1:**
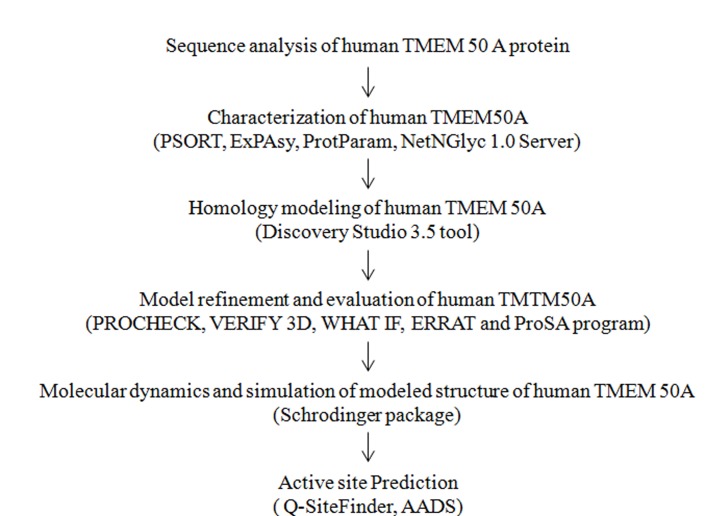
A workflow of modeling and molecular dynamics simulation for human TMEM 50A encoded by the TMEM 50A gene.

**Figure 2:**
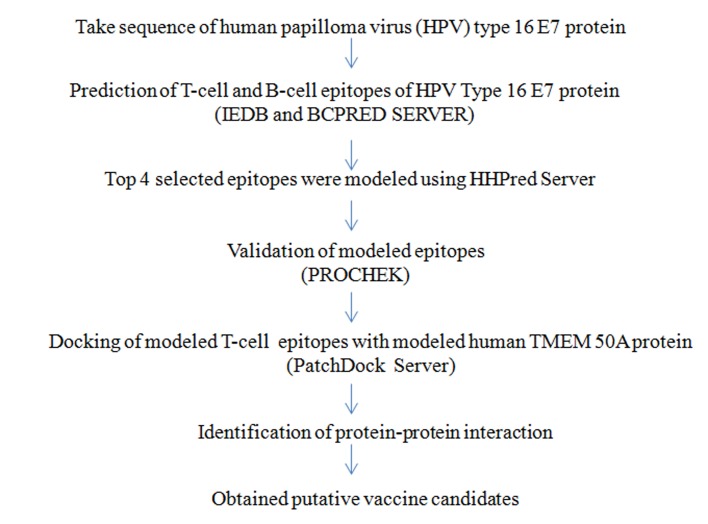
A workflow for linear epitope prediction

The workflow for analysis of modeling and dynamics simulation of human TMEM 50A (Figure 1) and generation of putative linear epitopes (Figure 2) are given.

### HPV 16 E7 Protein sequence retrieval

The 98 residues HPV type 16 E7 protein was downloaded from the Protein sequence database of NCBI (NCBI gene ID: 1489079 and NCBI protein id: 9627105).

### TMEM 50A sequence

The 157 residues human Trans membrane (TMEM) protein sequence was downloaded from the protein sequence database of NCBI (Swiss Prot ID O95807). Searching the Protein Data Bank (PDB) using BLASTP [9] identified the structural homolog of the TMEM 50A protein.

### Analysis of TMEM 50A

TMHMM 2.0 (http://www.cbs.dtu.dk/services/TMHMM-2.0/) and Target-P 1.1 server (http://www.cbs.dtu.dk/services/ TargetP) were used to predict the transmembrane helices in TMEM 50A. Sub-cellular localization of the protein using amino acid composition was completed using PSORT II (http://psort.hgc.jp/cgi-bin/runpsort.pl). NetNGlyc 1.0 Server (http://www.cbs.dtu.dk/services/ NetNGlyc/) predicted NGlycosylation sites using an artificial neural networks (ANN) model. Physiochemical properties such as molecular weight, theoretical pI, total number of negatively (Asp+Glu) and positively (Arg+Lys) charged residues, extinction coefficients, instability index, aliphatic indexed grand average of hydropathicity (GRAVY) of the mature protein were computed using Expasy׳s Prot pram proteomics server.

### Homology modeling for TMEM 50A

A structural model of TMEM 50A was generated using the template (PDB ID: 3um7A) with Discovery Studio Modeler 3.5 (http://www.accelrys.com) (see Table 1 for details).

### Model refinement and evaluation

The SAV server, which include various tools such as WHATIF [10], PROCHECK [11], PROVE, ERRAT [12], VERIFY-3D [13] and ProSA was used for model refinement. The stereo chemical quality and accuracy of the predicted models was evaluated with PROCHECK by Ramachandran plot analysis [14]. The best model was selected on the basis of overall G-factor, number of residues in core, allowed, generously allowed and disallowed regions. The selected model was further analyzed by VERIFY 3D, WHAT IF and ERRAT programme. ProSA was used to display Z-scores, energy plots and visualized with Discovery studio Modeler 3.5.

### The molecular dynamic simulation of TMEM 50A

The Schrodinger software package (https://www.schrodinger .com) was used for the molecular dynamics simulation of the predicted TMEM 50A model.

### Active sites prediction of TMEM 50A

We used Q-SiteFinder to locate binding site. Q-SiteFinder (http://www.bioinformatics.leeds.ac.uk/qsitefinder) uses the interaction energy between the protein and a simple van der Waals probe to locate energetically favorable binding sites. It also generates predicted sites with the lowest average volumes of the protein. The Active site finder (AADS) [15] was used to identify all cavities in a protein and scores them based on the physicochemical properties of functional groups lining the cavities in the protein. It is a freely available at http://www.scfbio-iitd.res.in/dock/ActiveSite_new.jsp.

### Computational epitope prediction for HPV type 16 E7 proteins

The FASTA amino acid sequence of HPV type16 E7 protein was retrieved from Swiss-Prot (Accession No. P03129) at http: //expasy.org/sprot/. The protein sequence of HPV 16 E7 was submitted to BCPREDS server (http://ailab.cs.iastate.edu/bcpreds/) and ABCPred online tool available at (http://www.imtech.res.in/raghava/abcpred) [16] for B-cell epitope prediction. IEDB analysis tool was used (http://tools.immuneepitope.org) was used for T-cell epitope prediction. This server covers a broad range of tools facilitating the prediction of new B-cell and T-cell epitopes in proteins of interest, and tools for the analysis of epitopes sets collected from within the IEDB. Alleles considered for prediction in this study include HLA-A*01:01, HLA-A*02:01, HLA-DRB1*01:02 and HLADRB1* 01:01.

### Docking of Human TMEM 50A (human Transmembrane Protein) with predicted T-cell epitopes of HPV type 16 E7 antigens

The 3D structure prediction of all the HLA allele specific predicted epitopes was completed using the HHPred server (http://toolkit.tuebingen.mpg.de/hhpred). The antigen-antibody docking of modeled epitopes of HPV and human protein TMEM 50A were completed using the PatchDock server (http://bioinfo3d.cs.tau.ac.il/PatchDock). PatchDock is a geometry-based molecular docking algorithm, which is aimed at finding docking transformation, Global energy, attractive vdW, repulsive vdW, hydrogen bond and ACE that yield good molecular shape complementarity [17]. The top ranked predicted epitopes were also docked with TMEM 50A (receptor) human Trans membrane protein model. The epitope-receptor docked complex were further refined using the FireDock program (http://bioinfo3d.cs.tau.ac.il/FireDock/). FireDock is an efficient method for refinement and re-scoring of rigid-body proteinprotein docking solutions [18]. The docked complexes based on their energy scores (kJ/mole), a comparative analysis was carried out and visualized with the help of Discovery studio 3.5.

### Prediction of protein-protein interaction of docked molecules

InterProSurf was used for protein-protein interactions studies of the docked molecules, which investigated the role of hydrogen bond formation, hydrophobic residues and overall electrostatics with total accessible surface area of the complex protein molecules, which includes polar area/energy and apolar area/energy. InterProSurf analyzed each chain within the complex and provides interface residues, interface area of each residue and a change in the surface area of each residue upon complex formation. It is freely available at http://curie.utmb.edu/prosurf.html [19].

## Results and Discussion

### HPV 16 E7 Protein sequence Analysis

Analysis of HPV 16 E7 ProtPram provides molecular weight (11.02 kD), theoretical pI (4.20), protein iso-electric point as pH 3.97 and total number of negatively charged residues (Asp + Glu) as 19, total number of positively charged residues (Arg + Lys) as five and ext. coefficient was 6335. The estimated half-life is: 30 hours (mammalian reticulocytes, in vitro) >20 hours (yeast, in vivo), total number of atoms found as 1499 and instability index second was computed to be 63.00. This classified the protein HPV type 16 E7 as stable and it also measured aliphatic index as 78.57 and grand average of hydropathicity (GRAVY) as 0.405.

### Characterization of TMEM 50A (Human protein)

TMHMM server 2.0 (http://www.cbs.dtu.dk/services/TMHMM/) and Target-P 1.1 server (http://www.cbs.dtu.dk/services/ TargetP/) [20] predicted the presence of four transmembrane domains within the signal peptide, which may be required to direct the protein to secretary pathway. Physiological properties found as molecular weight (17400Da), basal is electronic point (5.57), 17388.70 (monoisotopic mass) and the result of K-NN prediction using PSORT- II gives percentage of plasma-membrane (43.5%), endoplasmic reticulum (26.1%), vacuolar (17.4%), Golgi bodies (4.3%), mitochondrial (4.3%). Post-translation modification using PhosphoSitePlus predicted two serine phosphorylation sites found at amino acid 82 and 84 residue, one possible N-linked glycosylation site located at amino acid 74, and one possible tyrosine phosphorylation site is found. Expasy’s ProtPram proteomics server predicted the instability index (II) was computed to be 28.66, aliphatic index score as 95.10 and grand average of hydropathicity (GRAVY) as 0.580 which classified the protein as stable.

### Model refinement and evaluation of Human TMEM 50A

**Figure 3:**
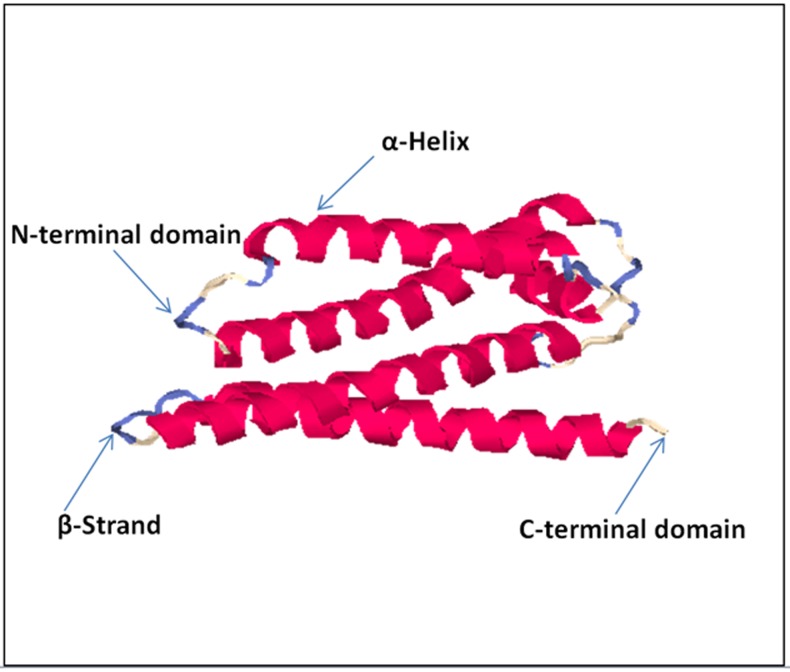
Predicted TMEM 50A model visualized using Discovery Studio 3.5.

PROCHECK server provided Ramachandran plot analysis of the predicted model. The best model in terms of stereo chemical quality showed Overall G-factor value of -0.38 which indicates that geometry of the model corresponds to high probability confirmation with 85.8% Residues in most favored regions [A, B, L], 9.0% residues in additional allowed regions [a, b, l, p], 2.2% residues in generously allowed regions [~a, ~b, ~l, ~p] and 3.0% of the residues was present in the disallowed region of the plot. Verify 3D analysis revealed that 80% of the residues had an average 3D-1D score of <0.2, predicting that the model is compatible with its sequence. The amino acid environment was evaluated using ERRAT plots, which assess the distribution of different types of atoms with respect to one another in the protein model and is used for making decision about its reliability. ERRAT showed an overall quality factor of 31.544, a result expected for crystallographic models with resolution>2.5A. Bfactor analysis is done with WHAT IF server reflected the mobility or flexibility of various parts of the molecule. Averaged B-factor deviation for protein backbone was 0.082 (Z score mean) and averaged standard deviation was 2.011. Since average deviation value was less than standard deviation, so it reflected a good quality model Figure 3.

### Molecular dynamic simulation of TMEM 50A

**Figure 4:**
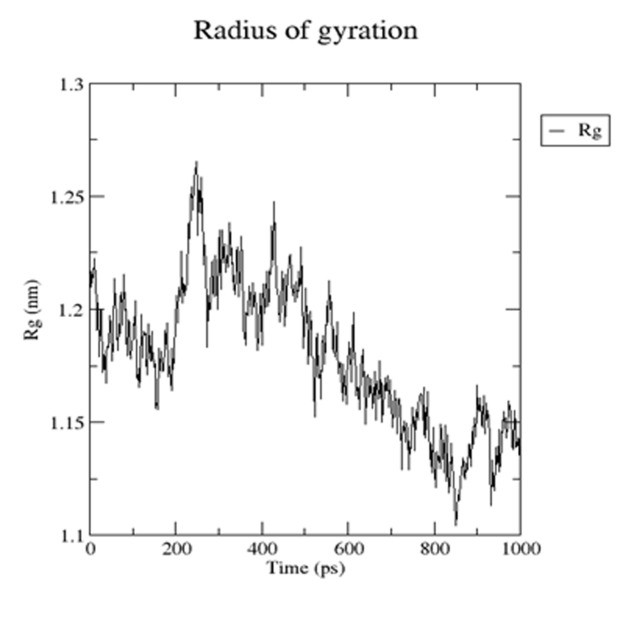
Radius of gyration (Rg) for human TMEM 50A protein.

**Figure 5:**
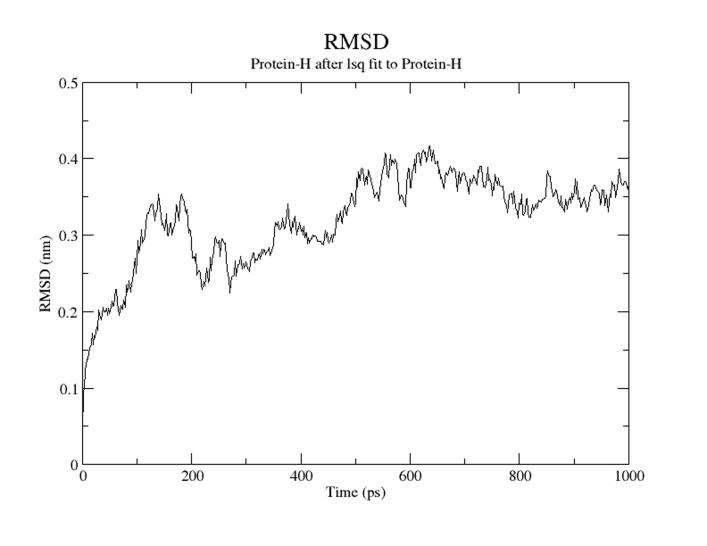
RMSD of the human TMEM 50A protein model for 10 ns.

**Figure 6:**
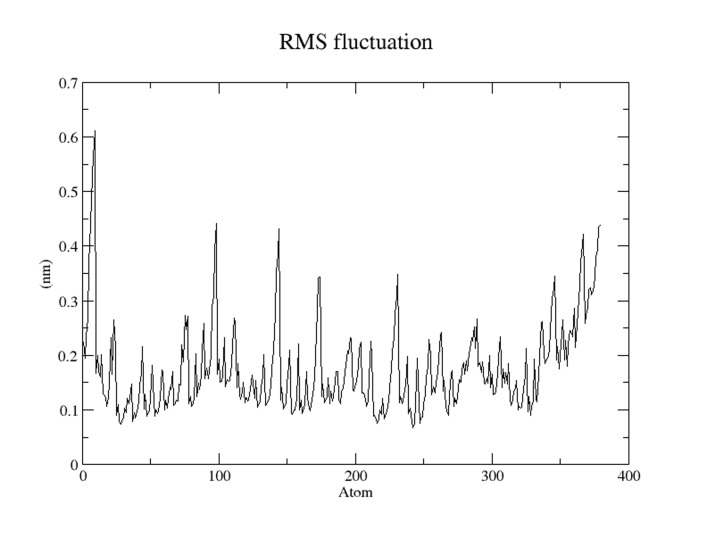
RMS fluctuation at 10 ns for the TMEM 50A model. Xaxis represents the time in picoseconds and Y-axis represents RMSD in nm unit.

The Molecular dynamic stability of TMEM 50A protein has been established by performing the molecular dynamic simulation study using Schrodinger software packages showed the energy of the molecule, which was found constant throughout the time period of simulation. In Figure 4 the radius of gyration increases in between 200 to 400 ps time but after 400 ps it decreases up to 1000 ps and finally omens almost constant for rest of the duration of the simulation. This suggests that the Human TMEM 50A protein model has a compact structure, which provides the required stability to the molecule. In Figure 5 the root mean square deviation (RMSD) during the simulation was increasing in the beginning and at 600 ps it showed highest peak value but after that it becomes almost constant for rest of the duration of the simulation. This suggests that the Human TMEM 50Amodel has lesser RMSD for Its backbone and it also has less flexibility, indicating that this protein has a stable dynamic behavior structure. In Figure 6 the root mean square (RMS) fluctuations were very less because most of the atoms were free from the RMS fluctuations but some atoms showed this at C and N terminal due to the presence of loop regions of modeled TMEM 50A.

### Active site prediction result

Q-SiteFinder predicted site volume and protein volume in cubic Angstrom with the minimum and maximum co-ordinates [21] and Active site finder (AADS) generated top 15 cavity points for TMEM 50A each pocket was represented by a single cavity point. Then it was sorted into cavities in the descending order of their volumes. The cavities generated in protein along with the Cartesian coordinates of the cavities are shown in Table 2.

### MHC Class I binding epitope prediction

Table 3 and Table 4 shows the predicted T-cell MHC class-I, epitopes for alleles HLA-A*01:01 and HLA-A*02:01 respectively. These small peptides (epitopes) were predicted by the IEDB analysis tool and ranked according to their consensus percentile rank. These percentile ranks refer to the percentage of rank out of one million random peptides from swiss-prot proteins [22]. In this the tap scores of the predicted epitopes were measured as local sequence to structure fitness based on torsion angle propensities normalized against the global minimum and maximum. In Table 3 top four epitopes MHGDTPTLHE, HGDTPTLHEY, GDTPTLHEYM and DTPTLHEYML were selected for docking with receptor protein. While In the Table 4 the score rank of ANN and SMM were shown in the units of IC50 (nM), which demonstrated that better binders have a low value of ANN and SMM rank. In this data include measured binding affinities for a total of 15 ranked epitopes for allele HLA-A*02:01. The top ranked epitopes YMLDLQPETT, DLLMGTLGIV, LLMGTLGIVC and TLEDLLMGTL was selected for epitope modeling and docking process. In particular a value of 500 (IC50) is often used as the threshold between binders and non-binders. So, peptides sequence YMLDLQPETT, DLLMGTLGIV, LLMGTLGIVC and LLMGTLGIVC represents highest affinity for binding with receptor proteins.

### MHC class II binding epitope prediction for allele HLA DRB1*01:01

Table 5 and Table 6 shows peptides for MHC Class-II alleles HLA-DRB1*01:01 and HLA-DRB1*01:02 predicted by IEDB analysis tool which were ranked according to their consensus percentile rank. In these predicted epitopes HVDIRTLEDLLMGTL, IRTLEDLLMGTLGIV, VDIRTLEDLLMGTLG and RTLEDLLMGTLGIVC were selected for docking with TMEM 50A. In Table 6, for each peptide, a percentile rank for Sturniolo method was generated by comparing the peptides score against the score of five million random isomers selected from Swiss-Prot database. It should be noted that small numbered percentile rank indicates high affinity. So the peptide sequences DLLMGTLGIVCPICS, LLMGTLGIVCPICSQ, LMGTLGIVCPICSQK and MGTLGIVCPICSQKP represents high affinity towards receptor proteins because these contained same percentile score and Sturniolo rank of 0.78.

### Docking of MHC Class-I epitopes and TMEM 50A

Top ranked predicted epitopes for different alleles of MHC Class I was docked with human TMEM 50A receptor using Patch Dock server. The results have been shown in the Table 7 and Table 8. The outputs of these tables were ranked by the global energy value, which was represented as the binding energy of the docked structures. ACE was used to calculate the free energies of transferring side-chains from protein interior into water. An ACE provides a reasonably accurate and rapidly calculated salvation component of free energy, and thus makes possible a range of docking, design and protein folding calculations [23]. Besides this, attractive Vdw and repulsive Vdw show the contribution of the Van der Waals forces to the global binding energy. In Table 7, docked structure of modeled epitope MHGDTPTLHE of MHC class-I (HLA-A*01:01) and TMEM 50A showed global energy as - 16.45 while other modeled epitopes HGDTPTLHEY and DTPTLHEYML of this class showed global energy as -60.15 and - 40.25 with TMEM 50A receptor protein while in Table 8 docked structure of modeled epitope YMLDLQPETT of MHC class I (HLA-A*02:01) and TMEM 50A showed global energy as -60.59.

### Docking of MHC class-II epitopes and TMEM 50A

**Figure 7:**
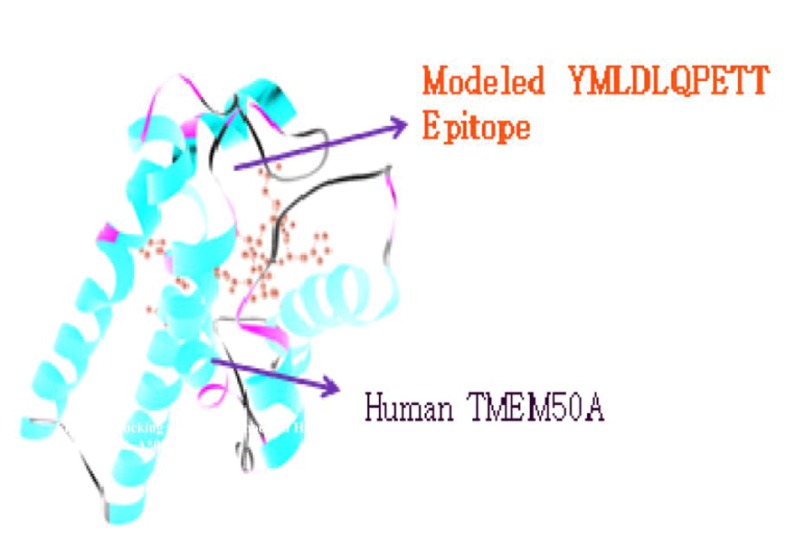
Molecular docking of YMLDLQPETT (E7 antigen) peptide with predicted HLA-A*02:01 specificity to human TMEM50A

Top ranked predicted epitopes for different alleles of MHC class- II were docked with human TMEM 50A receptor using Patch Dock server. The results are shown in the Table 9 and Table 10. The top ranked predicted MHC class-I and MHC class-II modeled epitopes were docked with their respective receptor Human TMEM 50 A. HVDIRTLEDLLMGTL docked with human TMEM 50 A showed global energy as -38.45 while another docked molecule IRTLEDLLMGTLGIV, RTLEDLLMGTLGIVC and VDIRTLEDLLMGTLG contained global energy as -66.57, - 9.25 and -9.25. In Table 10 docked molecule DLLMGTLGIVCPICS and human TMEM 50A have global energy as -47.59 while another docked molecules LLMGTLGIVCPICSQ, LMGTLGIVCPICSQK and MGTLGIVCPICSQKP have global energy as-40.36, -54.74 and -39.75 respectively. The modeled epitope DLLMGTLGIVCPICS.pdb was choosen for docking with human TMEM 50 A protein because it has -11.27 as atomic contact energy (ACE) and global energy as -47.59 was given suitable binding affinity to with human protein. In Figure 7 docked molecule showed binding of modeled epitope YMLDLQPETT of MHC Class-I (HLA-A*02:01) with human TMEM 50A receptor protein with global energy -60.59, attractive Vdw -24.24 and repulsive Vdw 26.54. In this, the contribution of the atomic contact energy (ACE) and hydrogen bonds (HB) energy to the global binding energy was found as -9.33 and -1.39. After this InterProSurf server predicts the total accessible surface area of this docked structures as 1568.43 in which polar area/energy was 610.73 and apolar area/energy was 957.70 with 1.4 prob radiuses.

**Figure 8:**
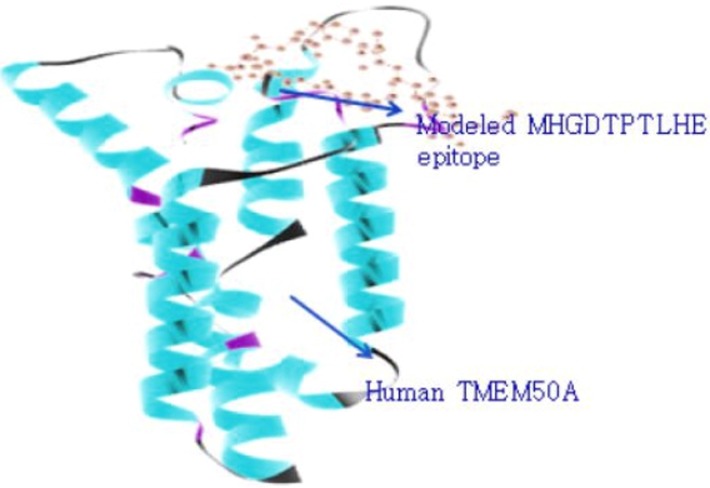
Molecular docking of MHGDTPTLHE (E7 antigen) peptide with predicted HLA-A*01:01 specificity to human TMEM50A.

**Figure 9:**
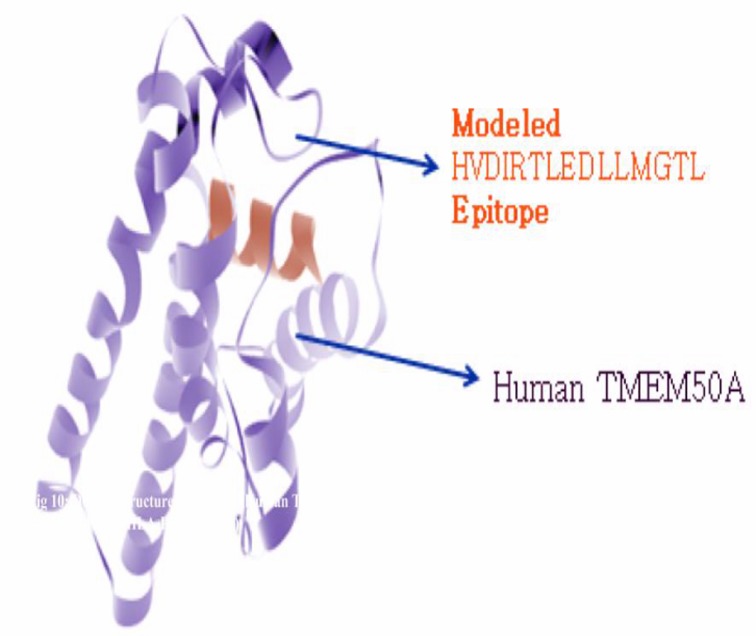
Molecular docking of HVDIRTLEDLLMGTL (E7 antigen) with predicted HLA-DRB1*01:01 specificity to human TMEM50A.

In Figure 8 docked molecule showed binding of modeled epitope MHGDTPTLHE of MHC class-I (HLA-A*01:01) to with human TMEM 50A with global energy -16.45, attractive Vdw -26.45, repulsive Vdw 10.97, ACE -0.99, HBs -0.52 which gave total accessible surface area of this docked structure as 11286.18 in which polar energy was 2870.52 and apolar energy was 8415.65 with 1.4 prob radiuses. In Figure 9 docked molecules showed binding of modeled epitope DLLMGTLGIVCPICS of MHC- II (HLA-DRB1*01:02) to with human TMEM 50A receptor protein with global energy -47.59, attractive vdW -29.83 and repulsive vdW 7.75, which gave more accurate vaccine candidate structure in comparison to rest of the molecules. In this the contribution of the atomic contact energy (ACE) and hydrogen bonds (HBs) energy to the global binding energy was found as -11.27 and - 0.95.

**Figure 10:**
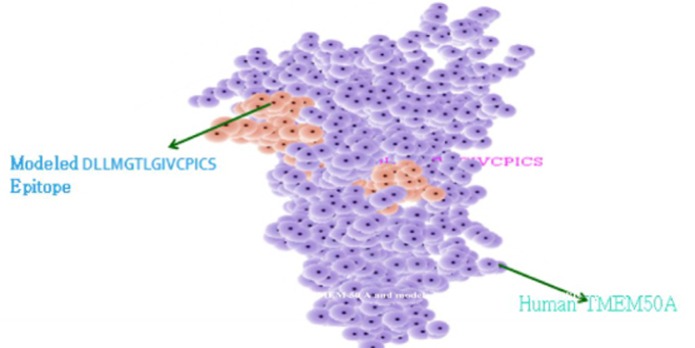
Molecular docking of DLLMGTLGIVCPICS (E7 antigen) with predicted HLA-DRB1*01:02 specificity to human TMEM 50A.

Beside this, InterProSurf server predicted the total accessible surface area of the docked structure as 11286.18 in which polar area/energy were 2870.52 and apolar area/energy was 8415.65 with 1.4prob radiuses. It also predicted the total number of surface atoms as 859 and total buried atoms were 371. In Figure 10, docked molecule showed binding of modeled epitope HVDIRTLEDLLMGTL of MHC Class-II (HLA-DRB1*01:01) to with human TMEM 50A receptor protein. ) In which modeled epitope protein showed in blue color and human TMEM 50 A shown in green color visualized with Discovery Studio 3.5 client tool with global energy -38.45, attractive Vdw -26.15 and repulsive Vdw 27.01 which gave more accurate vaccine candidate structure in comparison to rest of the molecules. In this the contribution of the atomic contact energy (ACE) and hydrogen bonds (HBs) energy to the global binding energy was found as - 9.33 and -1.39. After this InterProSurf server predicted the total accessible surface area of this docked structure as 9569.38 in which polar area/energy was 2740.68 and apolar area/energy was 6828.70 with 1.4 prob radiuses. In this total surface atoms and number of buried atoms were found as 775 and 455. Amino acid residues found at protein interface were GLU, ARG and CYS at residue number 6, 9 and 10 with complex area 46.11, 74.52 and 98.17.

## Conclusion

We document the predicted binding of linear peptide epitopes YMLDLQPETT (HLA-A*02:01), MHGDTPTLHE.HLA-A*01:01, DLLMGTLGIVCPICSHLA-DRB1*01:02 and HVDIRTLEDLLMGTL (HLA-DRB1*01:01) in HPV type 16 E7 antigen with the human TMEM 50 A protein structural model. The data documented in this study finds application in development of components towards HPV associated disease prevention.

## Figures and Tables

**Table 1:** Top 10 templates selected for homology modeling of TMEM 50A.

Rank	Pdb hit	Iden 1	Iden 2	Cov.	Norm Z-Score
1	3um7A	0.11	0.13	0.64	1.2
2	2kseA	0.2	0.1	0.41	1.17
3	4gbyA	0.17	0.2	0.96	0.73
4	3kdpA	0.2	0.17	0.44	0.52
5	1f4pA	0.16	0.2	0.83	0.79
6	4aKfA	0.09	0.17	0.94	0.73
7	2wscA	0.09	0.19	0.68	1.06
8	3KdpA	0.21	0.17	0.45	0.96
9	3lqhA	0.1	0.15	0.98	0.47
10	1kfyC	0.14	0.12	0.54	0.6

Note: In this Iden 1 shows the percentage sequence identity of the templates in the threading aligned region and Ident 2 is the percentage sequence identity of the whole template chains with the TMEM 50A sequence and Norm Z-score is the normalized Z-score of the threading alignments. Alignment with a Normalized Z-score > 1 means a good alignment.					

**Table 2:** Coordinates of cavity points generated by active site finder in TMEM 50A along with an approximate volume in the respective cavity.

S. No.	Predicted cavity on surface of protein TMEM 50A	Cavity Point X-axis	Cavity Point Y-axis	Cavity Point Z-axis	Volume of the Cavity
1	Cavity_1_INQFMGLKWVRSACPDEYT	1.081	5.287	-8.321	1041
2	Cavity_2_FVLWAISCMGNEYDHQT	7.059	4.219	-3.115	1027
3	Cavity_3_FILAMWCGSTVEYHDQR	6.825	6.48	4.059	998
4	Cavity_4_QVGYIANFKLMWSCPHD	-6.961	-7.723	-7.551	877
5	Cavity_5_FMLSIACWNGTVYHED	1.28	-0.354	-0.236	764
6	Cavity_6_NIFMGLKSACTWVYH	-6.463	-0.296	3.116	752
7	Cavity_7_MFLSAICNTGVHYKDW	0.922	-6.497	1.395	742
8	Cavity_8_AGIVQYFNKLMEWSPTD	-5.552	-5.106	-16.664	701
9	Cavity_9_QVGIANYFKLMWSC	-9.944	-6.388	-5.614	621
10	Cavity_10_IFGLKARTVMWES	-7.522	10.031	2.421	578

**Table 3:** Prediction of linear epitopes in HPV 16 E7with HLA-A*01:01 using NetMHCpan-IEDB

S. No	Position Start-End	Pep Length	Sequence	Proteasome Score	TAP Score	MHC Score	Processing Score	Total Score	MHC IC50 [nM]
1	1:1-10	10	MHGDTPTLHE	1.15	-0.76	-4.58	0.4	-4.19	38150.11
2	1:2-11	10	HGDTPTLHEY	1.46	1.05	-2.35	2.51	0.16	226.04
3	1:3-12	10	GDTPTLHEYM	1.09	-0.11	-4.52	0.98	-3.53	32787.64
4	1:4-13	10	DTPTLHEYML	1.54	0.25	-4.33	1.79	-2.54	21500.59
5	1:5-14	10	TPTLHEYMLD	0.75	-1.01	-4.62	-0.25	-4.87	41599.44
6	1:6-15	10	PTLHEYMLDL	1.17	0.21	-4.3	1.38	-2.92	19932.31
7	1:7-16	10	TLHEYMLDLQ	0.65	-0.1	-4.48	0.55	-3.93	30396.07
8	1:8-17	10	LHEYMLDLQP	0.74	-0.01	-4.58	1.38	-3.86	38150.11
9	1:9-18	10	HEYMLDLQPE	0.66	-0.67	-4.65	0.55	-4.66	44389.61
10	1:10-19	10	EYMLDLQPET	1.22	-0.26	-4.6	0.73	-3.63	39408.75
11	1:11-20	10	YMLDLQPETT	1.08	-0.33	-4.52	-0.01	-3.76	32787.64
12	1:12-21	10	MLDLQPETTD	0.91	-0.86	-4.31	0.96	-4.22	20589.91
13	1:13-22	10	LDLQPETTDL	1.28	0.31	-4.62	0.75	-3.03	41599.44
14	1:14-23	10	DLQPETTDLY	1.16	1.15	-3.45	0.05	-1.14	2812.19
15	1:15-24	10	LQPETTDLYC	0.99	-0.03	-4.53	1.59	-3.56	33504.88

**Table 4:** Prediction of linear epitopes in HPV 16 E7with HLA-A*02:01 using ANN and SMM methods

S. No.	Position Start-End	Peptide Length	Sequence	Percentile Rank	ANN IC50 (nM)	ANN Rank	SMM IC50 (nM)	SMM Rank
1	1:11-20	10	YMLDLQPETT	0.55	22.75	0.3	74.55	0.8
2	1:81-90	10	DLLMGTLGIV	2.1	398.58	2.8	125.23	1.4
3	1:82-91	10	LLMGTLGIVC	3.45	590.13	3.4	351.48	3.5
4	1:77-86	10	LLMGTLGIVC	3.85	429.65	2.9	509.08	4.8
5	1:78-87	10	TLEDLLMGTL	4.55	485.03	3.1	672.48	6
6	1:86-95	10	TLGIVCPICS	13.45	7138.72	12.1	2481.73	14.8
7	1:7-16	10	TLHEYMLDLQ	14.15	11455.59	16.6	1720.17	11.7
8	1:84-93	10	MGTLGIVCPI	14.75	9570.08	14.6	2506.35	14.9
9	1:83-92	10	LMGTLGIVCP	15.55	10487.31	15.6	2660.67	15.5
10	1:63-72	10	STLRLCVQST	16.3	6405.4	11.4	4590.33	21.2
11	1:12-21	10	MLDLQPETTD	18.55	17783.65	23.9	2062.92	13.2
12	1:65-74	10	LRLCVQSTHV	18.75	14280.54	19.7	3387.63	17.8
13	1:19-28	10	TTDLYCYEQL	20.4	5097.15	10	9475.89	30.8
14	1:6-15	10	PTLHEYMLDL	20.55	11946.61	17.1	5770.05	24
15	1:21-30	10	DLYCYEQLND	21.45	20500.33	27.6	2623.49	15.3

**Table 5:** Prediction of linear epitopes in HPV 16 E7 protein with HLA-DRB1*01:01 using IEDB Consensus tool

S. No.	Position Start-End	Peptide length	Peptide Sequence	Percentile Rank	Comb. Lib IC50 (nM)	SMM IC50 (nM)	SMM Align Rank
1	73-87	10	HVDIRTLEDLLMGTL	18.67	4583.61	75	14.01
2	76-90	10	IRTLEDLLMGTLGIV	21.79	33.35	111	19.12
3	74-88	10	VDIRTLEDLLMGTLG	23.61	4583.61	73	13.7
4	77-91	10	RTLEDLLMGTLGIVC	23.84	33.35	118	20.03
5	72-86	10	THVDIRTLEDLLMGT	23.88	26863.95	92	16.56
6	Sep-23	10	HEYMLDLQPETTDLY	24.27	98373.03	154	24.27
7	Oct-24	10	EYMLDLQPETTDLYC	25.18	98373.03	163	25.18
8	Aug-22	10	LHEYMLDLQPETTDL	25.68	98373.03	159	24.78
9	75-89	10	DIRTLEDLLMGTLGI	26.02	33.35	124	20.8
10	78-92	10	TLEDLLMGTLGIVCP	26.47	33.35	141	22.88
11	71-85	10	STHVDIRTLEDLLMG	28.88	69.54	97	17.28
12	79-93	10	LEDLLMGTLGIVCPI	29.89	33.35	144	23.22
13	62-76	10	DSTLRLCVQSTHVDI	31.65	47.03	238	31.51
14	61-75	10	CDSTLRLCVQSTHVD	32.83	47.03	257	32.83
15	63-77	10	STLRLCVQSTHVDIR	32.97	47.03	259	32.97

**Table 6:** Prediction of epitopes in E7 with HLA-DRB1*01:02 using IEDB-Sturniolo method

S. No.	Position Start-End	Peptide Sequence	Percentile Rank	Sturniolo Core	Sturniolo Score	Sturniolo Rank
1	1:81-95	DLLMGTLGIVCPICS	0.78	LGIVCPICS	2.05	0.78
2	1:82-96	LLMGTLGIVCPICSQ	0.78	LGIVCPICS	2.05	0.78
3	1:83-97	LMGTLGIVCPICSQK	0.78	LGIVCPICS	2.05	0.78
4	1:84-98	MGTLGIVCPICSQKP	0.78	LGIVCPICS	2.05	0.78
5	1:70-84	QSTHVDIRTLEDLLM	3.28	IRTLEDLLM	0.78	3.28
6	1:71-85	STHVDIRTLEDLLMG	3.28	IRTLEDLLM	0.78	3.28
7	1:72-86	THVDIRTLEDLLMGT	3.28	IRTLEDLLM	0.78	3.28
8	1:73-87	HVDIRTLEDLLMGTL	3.28	IRTLEDLLM	0.78	3.28
9	1:74-88	VDIRTLEDLLMGTLG	3.28	IRTLEDLLM	0.78	3.28
10	1:75-89	DIRTLEDLLMGTLGI	3.28	IRTLEDLLM	0.78	3.28
11	1:76-90	IRTLEDLLMGTLGIV	3.28	IRTLEDLLM	0.78	3.28
12	1:77-91	RTLEDLLMGTLGIVC	3.65	LMGTLGIVC	0.6	3.65
13	1:78-92	TLEDLLMGTLGIVCP	3.65	LMGTLGIVC	0.6	3.65
14	1:79-93	LEDLLMGTLGIVCPI	3.65	LMGTLGIVC	0.6	3.65
15	1:80-94	EDLLMGTLGIVCPIC	3.65	LMGTLGIVC	0.6	3.65

**Table 7:** Docked energy score using FireDock for HLA-A*01:01 specific epitopes with Human TMEM 50 A protein.

Epitope	Global nergy (KJ/mol)	Attractive vdW	Repulsive vdW	ACE (atomic contact energy)	HB (hydrogen and disulphide bonds)	Transformation (ligand transformation after refinement)
MHGDTPTLHE	-16.45	-26.45	10.97	-0.99	-0.52	0.461751 0.356073 -1.483984 -32.507160 6.359675 -58.814320
HGDTPTLHEY	-60.15	-37.44	50	-17.29	-1.06	1.650077 0.501054 1.488182 0.437999 -33.281693 19.930298
DTPTLHEYML	-40.25	-35.06	11.92	-5.85	-5.27	0.125927 -1.041521 -1.280155 -8.511353 27.405537 21.995085

**Table 8:** Docked energy score using FireDock for HLA-A*02:01 specific epitopes with Human TMEM 50 A protein.

Epitope	Global Energy KJ/mol	Attractive vdW	Repulsive vdW	ACE	HB (Hydrogen and Disulphide bond)	Transformation (ligand transformation after refinement)
YMLDLQPETT	-60.59	-24.24	6.54	-15.33	-1.68	-1.799130 0.078302 -0.746196 -56.133747 -16.896145 24.083170
DLLMGTLGIV	-55.57	-28.15	11.13	-14.56	-3.41	1.133256 -0.037606 -0.816487 10.963738 -0.143226 3.577083
LLMGTLGIVC	-55.24	-24.39	3.4	-11.4	-2.46	0.720709 -0.175391 -1.310262 17.513165 -2.957317 1.888661
RTLEDLLMGT	-55.16	-22.14	2.12	-9.65	-1.2	-0.184480 0.320455 0.783296 9.089859 16.816320 -5.468778

**Table 9:** Docking using FireDock for HLA-DRB1*01:01 specific epitopes with human MEM 50A

Epitope	Global Energy (KJ/mol)	Attractive Vdw	Repulsive Vdw	ACE	HB (hydrogen and disulphide bonds)	Transformation (ligand transformation after refinement)
HVDIRTLEDLLMGTL	-38.45	-26.15	27.01	-9.33	-1.39	0.699969 -0.397265 -1.029715 -1.292001 14.140763 2.199753
IRTLEDLLMGTLGIV	-66.57	-36.11	52.99	-33.14	-1.22	1.075338 -0.722708 -2.897819 -1.582976 -2.212364 -7.311763
RTLEDLLMGTLGIVC	-9.25	-19.25	7.71	4.96	-2.61	-2.138732 0.465165 2.407196 16.671505 20.182180 18.587845
VDIRTLEDLLMGTLG	-9.25	-19.25	7.71	4.96	-2.61	-2.138732 0.465165 2.407196 16.671505 20.182180 18.587845

**Table 10:** Comparison of parameters HLA allele specific peptides docked to TMEM 50 A

S. No.	Epitopes and alleles	HLA alleles	Global energy	Attractive Vdw	Repulsive Vdw	Total accessible surface area	Polar energy/Apolar energy	No. of residue at interface	Prob Radius
1	YMLDLQPETT	A*02:01	-60.59	-24.24	26.54	11286.18	2870.52/8415.65	LEU	1.4
2	MHGDTPTLHE	A*01:01	-16.45	-26.45	10.97	11286.18	2870.52/8415.65	LEU	1.4
3	DLLMGTLGIVCPICS.	DRB1*01:02	-47.59	-29.83	7.75	11286.18	2870.52/8415.65	CYS	1.4
4	HVDIRTLEDLLMGTL.	DRB1*01:01	-38.45	-26.15	27.01	9569.38	2740.68/6828.70	Glu, ARG and CYS	1.4
